# Total Laboratory Automation Versus Manual Processing in Urine Culture Inoculation and Interpretation: A Hospital Experience

**DOI:** 10.3390/diagnostics16101474

**Published:** 2026-05-13

**Authors:** Nabeel Alzahrani, Atheer Alghamdi, Anmar Yankasar, Nawal Alyami, Hoda Abanmi, Bassam Alwan, Lana Alzamil, Sameera Al Johani

**Affiliations:** 1 Department of Clinical Laboratory Sciences, College of Applied Medical Sciences, King Saud bin Abdulaziz University for Health Sciences, Riyadh 11481, Saudi Arabia; 2 King Abdullah International Medical Research Center, Riyadh 11481, Saudi Arabia; 3 Division of Microbiology, Department of Pathology and Laboratory Medicine, Ministry of the National Guard-Health Affairs, Riyadh 14611, Saudi Arabia; 4 Research and Innovation Unit, College of Applied Medical Sciences, King Saud bin Abdulaziz University for Health Sciences, Riyadh 11481, Saudi Arabia

**Keywords:** urine culture, clinical microbiology, workflow efficiency, total laboratory automation

## Abstract

**Background/Objectives**: Urine culture processing is labor-intensive and prone to operator-dependent variability. This study compared total laboratory automation (TLA; BD Kiestra™) with manual urine culture processing in terms of workflow efficiency, diagnostic performance, and operator variability. **Methods**: Three hundred midstream urine specimens were processed using manual and automated workflows, stratified by technologist experience (expert ≥10 years; non-expert <2 years) and shift. Metrics included setup time, cleanup time, and total staff time (TST). Colony-forming unit (CFU) recovery using 1 µL manual inoculation, 10 µL manual inoculation, and 10 µL TLA inoculation of 20 known positive specimens was compared. Diagnostic performance was assessed against the 1 µL manual reference using serially diluted specimens at a ≥10^5^ CFU/mL threshold. **Results**: TLA reduced the setup time from 10 min 10 s to 2 min 05 s for experts and from 13 min 37 s to 2 min 20 s for non-experts (79–83% reduction). TST decreased from 11 min 06 s to 2 min 30 s and from 14 min 37 s to 3 min 15 s, respectively (77–78% reduction). Cleanup time showed smaller reductions that did not reach statistical significance in the paired analysis. Manual processing showed greater operator-dependent variability, which TLA substantially reduced. CFU recovery was concordant in high-burden specimens, with method-dependent differences in routine diagnostic samples. **Conclusions**: TLA improves urine culture workflow efficiency, reduces operator-dependent variability, and shows concordant semi-quantitative performance compared to the standard manual reference within the limits of a proof-of-concept design, supporting its implementation to enhance consistency and throughput in clinical microbiology laboratories.

## 1. Introduction

Urinary tract infections (UTIs) are among the most common bacterial infections encountered in both community and hospital settings and are major contributors to antibiotic prescriptions and healthcare resource utilization [1]. Accurate and timely microbiological diagnosis of UTIs is critical for ensuring effective treatment, preventing complications, and supporting antimicrobial stewardship [2,3]. Culture-based diagnosis remains the gold standard for UTI detection; however, conventional manual workflows are time-consuming, labor-intensive, and susceptible to performance variability across technologists and shifts [4]. As the demand for faster and more consistent diagnostics increases, microbiology laboratories are increasingly challenged to modernize their workflows without compromising their analytical integrity [5,6].

Manual urine culture processing involves several operator-dependent steps that introduce variability in colony isolation, quantification, and interpretation [7]. Additionally, the manual approach places significant demands on laboratory personnel, limiting throughput and prolonging turnaround times (TATs), particularly during periods of high workload or staff shortages. As healthcare institutions seek scalable and reproducible solutions, total laboratory automation (TLA) has emerged as a promising innovation for addressing these limitations.

TLA systems such as the BD Kiestra^™^ and Copan WASPLab^®^ platforms automate essential stages of culture processing, including specimen inoculation, streaking, incubation, and high-resolution digital imaging [8,9]. These systems aim to standardize microbiology laboratory workflows, reduce hands-on time, and enhance reproducibility across operators [10]. Several studies have demonstrated that TLA improves laboratory efficiency, enhances consistency, and reduces turnaround times compared with manual workflows [5,9,11,12,13]. However, the impact of TLA on inter-operator variability and its diagnostic performance across different levels of technologist experience under real-world conditions requires further evaluation. This study was designed to assess the effect of total laboratory automation on pre-analytical workflow efficiency—specifically specimen setup, inoculation, and cleanup—rather than the full analytical or reporting workflow, and to compare its performance and consistency with conventional manual processing. By building on the current literature and exploring outcomes across operator experience levels, this study seeks to provide meaningful insights into the evolving practices of microbiology automation.

## 2. Materials and Methods

### 2.1. Study Design and Clinical Specimens

This comparative study was conducted at the Clinical Microbiology Laboratory of the Ministry of the National Guard Hospital, Riyadh, Saudi Arabia. A total of 300 midstream urine specimens submitted for routine culture were processed to evaluate the differences in workflow efficiency and diagnostic performance between manual and automated methods. To minimize sequence-related bias, the first 150 specimens were processed manually followed by automated processing, whereas the remaining 150 were processed in the reverse order (automated then manual). Specimen processing was conducted during three daily shifts (08:00–12:00, 13:00–15:00, and 15:00–18:00) to assess the potential temporal effects on the workflow. The laboratory operates 24 h per day; however, urine culture inoculation is only performed during the staffed daytime hours between 08:00 and 18:00. The ‘after 15:00′ shift in this study corresponds to the late-afternoon period (15:00–18:00). No inoculation or data collection occurred during the overnight period (18:00–08:00).

Urine samples were collected in sterile urinalysis cup kits (Becton, Dickinson and Company, Franklin Lakes, NJ, USA) with integrated transfer devices and transferred to 4 mL BD Vacutainer urinalysis tubes containing boric acid. Each sample was inoculated onto blood agar (BAP) and MacConkey agar (MAC) biplates (Watin, Riyadh, Saudi Arabia) and processed within 4 h of collection.

### 2.2. Manual Processing

Manual inoculation was performed by four certified microbiology laboratory scientists (MLSs): two experienced staff members (≥10 years of experience; ‘experts’) and two less experienced staff members (<2 years; ‘non-experts’). The same four technologists performed both manual and automated (TLA) workflows during the study, with each technologist contributing observations across multiple shifts. Each technologist × shift × workflow combination constituted one independent batch-level observation, yielding six batch-level observations per operator group per workflow. Using 10 µL calibrated loops, specimens were inoculated onto BAP and MAC biplates using a central streak followed by a zigzag pattern. The plates were incubated at 35–37 °C in 5% CO_2_ for 18–24 h. After incubation, colony morphology and growth were assessed manually, and the plates were either marked for further investigation or discarded if no growth was observed.

### 2.3. Automated Processing

Automated processing was performed using the BD Kiestra^™^ Total Laboratory Automation (TLA) system (Becton, Dickinson and Company, Franklin Lakes, NJ, USA), which includes the InoqulA and ReadA Compact modules. MLS staff loaded boric acid-containing vacuum tubes onto the BD Kiestra^™^ TLA track. The InoqulA module performed automated inoculation using magnetic bead-based streaking, and plates were transferred to integrated incubators configured to match the manual conditions (35–37 °C and 5% CO_2_) for 24 h. Digital images of each plate were acquired at 18 and 24 h post-inoculation. The MLS staff reviewed the images to assess growth and determine whether further workup or disposal was required. An on-site BD field engineer was available during working hours; however, no technical interventions or maintenance interruptions occurred during the study period.

### 2.4. Workflow Efficiency Assessment

Workflow efficiency was evaluated using predefined process metrics, including plate touches, setup time, cleanup time, and total staff time (TST). All measurements were recorded during routine batch processing by designated laboratory observers.

## 3. Plate Touches

Plate touches were defined as the number of discrete manual interactions with culture plates during the setup and inoculation phase, measured as a count per specimen, and excluding post-incubation handling and interpretation of growth.

- **Manual workflow:** Three discrete plate touches per specimen were counted: (1) plate labeling, (2) inoculation (central streak followed by zigzag pattern), and (3) transfer to the incubator. Any additional touches observed during processing were counted individually.
- **TLA workflow:** Zero plate touches per specimen were counted. Labeling, inoculation, and incubator transfer were performed by the automation system; operators only interacted with specimen tubes on the automation track during pre-analytical setup.

**Setup Time**

Setup time was defined as the duration of all pre-incubation specimen handling and preparation steps.

- **Manual workflow:** Included specimen preparation, labeling of culture media, selection and organization of consumables (e.g., calibrated loops and pipette tips), inoculation of culture plates, and arrangement of plates for incubation.
- **TLA workflow:** Included preparation of the automation system, including loading specimen racks, barcode scanning, verification of specimen order, placement of plates within the system, and initiation of automated inoculation.

**Cleanup Time**

Cleanup time was defined as the duration required to complete post-inoculation workspace and system-related tasks.

- **Manual workflow:** Included disposal of used consumables, cleaning of work surfaces, and restoration of workspace organization following inoculation.
- **TLA workflow:** Included removal of processed racks, cleaning of the loading area, and verification of system readiness for subsequent runs. Disposal of consumables was performed automatically and was not included.

**Total Staff Time (TST)**

Total staff time (TST) was defined as the sum of the setup time and cleanup time:

**TST = Setup Time + Cleanup Time**

For batch processing, the total time was normalized by dividing by the number of specimens processed per batch.

### 3.1. Quantification Accuracy Across Inoculation Methods

To evaluate the impact of inoculation volume on semi-quantitative urine culture results, a total of twenty urine specimens with confirmed bacterial growth were analyzed. Specimens were selected from the routine clinical workload of the Ministry of the National Guard Hospital’s microbiology laboratory based on their prior culture result obtained using the standard laboratory method (1 µL manual calibrated loop inoculation, performed and read by certified MLS staff as part of routine diagnostic practice). Ten specimens with a prior culture result of ≥10^5^ CFU/mL were classified as strong positives (Group C), and ten additional specimens with any positive prior culture result were randomly selected as routine-practice positives (Group D). The three inoculation methods compared in this experiment—1 µL manual loop, 10 µL manual loop, and 10 µL TLA bead-based inoculation—were compared to each other using the Friedman test; no single method served as the reference for this comparison, and sensitivity and specificity were not calculated in this experiment. Diagnostic performance metrics (sensitivity, specificity, PPV, and accuracy) were calculated separately in the Accuracy and Precision Testing experiment described below, using the 1 µL manual loop as the reference standard.

Each specimen was inoculated using three methods: manual inoculation with a 1 µL calibrated loop, manual inoculation with a 10 µL calibrated loop, and automated inoculation using the BD Kiestra™ TLA system’s 10 µL magnetic bead-based streaking mechanism. The plates were incubated according to the standard workflow described above, and colony counts were recorded to semi-quantitatively assess the recovery of the methods.

Each specimen was processed once using each inoculation method, resulting in a total of 60 observations. Groups C and D were used exclusively for this experiment and were distinct from the specimens included in the workflow efficiency analysis.

This comparison reflects differences in routine laboratory practice, where manual workflows commonly use a 1 µL loop, whereas TLA systems employ a fixed 10 µL of inoculum, which may influence semi-quantitative colony counts and interpretation of culture results.

### 3.2. Accuracy and Precision Testing

For diagnostic performance evaluation, a separate set of twenty urine specimens was prepared. Samples were standardized to McFarland 0.5 (~10^7^–10^8^ CFU/mL) and serially diluted to final concentrations of ≥10^5^, 10^5^–10^4^, 10^4^–10^3^, and <10^3^ CFU/mL.

All dilutions were processed using manual and TLA workflows. The 1 µL manual loop inoculation was used as the reference standard for urine culture quantification, consistent with standard semi-quantitative urine culture practice [14].

The diagnostic performance of the 10 µL manual and TLA methods was evaluated relative to this reference using the ≥10^5^ CFU/mL threshold for clinical positivity.

Performance metrics were calculated as follows:

Sensitivity = TP/(TP + FN);

Specificity = TN/(TN + FP);

Positive Predictive Value (PPV) = TP/(TP + FP);

Accuracy = (TP + TN)/(TP + TN + FN + FP).

Ninety-five percent confidence intervals for each performance metric were calculated using the Wilson score method.

### 3.3. Statistical Analysis

Descriptive statistics were presented as medians and interquartile ranges (IQRs) due to the nonparametric distribution of the data, with mean ± standard deviation (SD) reported alongside in Table 1 and Table 2 to provide a fuller description of the batch-level distributions. The complete per-batch dataset, comprising all 24 independent technologist × shift × workflow observations, is provided in Supplementary Table S3. Comparisons of setup time, cleanup time, and total staff time (TST) between manual and TLA workflows within each user group (expert and non-expert) were performed using the Wilcoxon signed-rank test (two-tailed, asymptotic), reflecting the paired nature of the batch-level measurements: each technologist × shift combination contributed one batch under each workflow, generating naturally paired observations. Comparisons between expert and non-expert users within each workflow were performed using the Mann–Whitney U test (two-tailed, asymptotic), as these comparisons involved different operators without natural pairing. For workflow timing analyses, the unit of observation was the batch. Each subgroup contributed six batch-level observations, with 25 specimens processed per batch.

For comparisons of paired measurements across multiple inoculation methods (1 µL manual loop, 10 µL manual loop, and automated inoculation using the BD Kiestra™ total laboratory automation system) for the same specimens, the Friedman test was applied. Where the Friedman test indicated a statistically significant overall difference, pairwise post hoc comparisons between inoculation methods were performed using the Wilcoxon signed-rank test with Bonferroni correction for three comparisons. The pairwise comparisons reported in Table 1 and Table 2 and Supplementary Tables S1A,B reflect a pre-specified analytic structure (workflow × operator × metric) that follows directly from the study design rather than an exploratory search across many tests. In this confirmatory framework, where each comparison addresses an a priori hypothesis embedded in the study’s stratification scheme, mechanical family-wise error-rate correction (e.g., Bonferroni or Benjamini–Hochberg) may discard genuine findings without a proportionate gain in inferential validity [15,16]. We therefore report uncorrected *p*-values for these pre-specified comparisons while explicitly flagging this analytic choice. Family-wise error-rate correction (Bonferroni) was applied to the post hoc pairwise comparisons that followed each Friedman test, where exploratory pairwise testing within a multi-arm design genuinely warranted control of false-positive rates.

Diagnostic performance metrics—including sensitivity, specificity, positive predictive value (PPV), and accuracy—were calculated relative to the 1 µL manual loop inoculation, which served as the reference standard for urine culture quantification in accordance with American Society for Microbiology manual semi-quantitative practices.

All statistical analyses were conducted using IBM SPSS Statistics version 31 (IBM Corp., Armonk, NY, USA) and Microsoft Excel (Microsoft Corp., Redmond, WA, USA). Statistical significance was defined as *p* < 0.05.

## 4. Results

**Figure 1:**
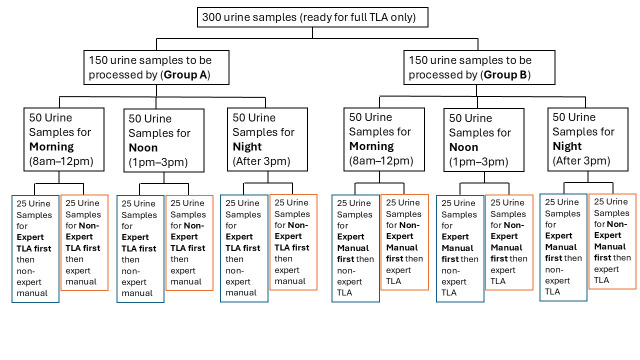
**Experimental design and sample allocation**. Three hundred urine specimens were divided into Group A (TLA followed by manual) and Group B (manual followed by TLA). Each group (*n* = 150) was subdivided by shift (morning, noon, and late afternoon) and technologist experience (expert vs. non-expert), with 25 specimens per subgroup and two processing-order permutations, enabling assessment of workflow efficiency and operator variability.

A total of 300 midstream urine specimens were included in this study and evenly divided into 6 groups of 50 samples each. All automated urine culture processing in this study was performed using the BD Kiestra™ Total Laboratory Automation (TLA) platform, which integrates automated inoculation, incubation, and high-resolution digital imaging modules. Group A specimens were processed first by TLA followed by manual plating, whereas Group B specimens were processed in the reverse order (manual first, then TLA). Each group was further subdivided by shift (morning: 8:00–12:00; noon: 13:00–15:00; and late afternoon: 15:00–18:00) and by technologist experience level (expert vs. non-expert). Within each subgroup, 25 specimens were processed under each of the two technologist permutations: expert followed by non-expert, and non-expert followed by expert (Figure 1). Workflow timing analyses were based on six batch-level observations per subgroup, with 25 specimens processed per batch. This stratified design allowed for the assessment of workflow efficiency and diagnostic performance across different processing sequences, time shifts, and operator expertise levels. In addition, this study evaluated setup time, cleanup time, total staff time (TST), and bacterial colony quantification using colony-forming units (CFUs) as a measure of recovery. Inoculation was performed using 1 µL and 10 µL calibrated loops for the manual methods, and fixed 10 µL magnetic bead-based inoculation for the BD Kiestra™ TLA platform. Diagnostic performance was assessed by calculating sensitivity, specificity, positive predictive value (PPV), and overall accuracy based on a clinical threshold of ≥10^5^ CFU/mL.

### 4.1. Comparison of Plate Touches Between Manual and TLA Workflows

In addition to time-based workflow metrics, the number of plate touches per specimen (count) was evaluated as a direct measure of manual interaction with culture plates (see Methods for the enumerated definition). Manual workflows required substantially more plate touches compared to TLA. Within the manual workflow, expert users generally demonstrated fewer plate touches than non-experts, although these differences were not consistent across all shifts.

When comparing workflows, TLA resulted in a marked reduction in plate touches for both expert and non-expert technologists across all shifts (*p* < 0.0001 in all comparisons; Table S1A). Within the manual workflow, expert users demonstrated fewer plate touches than non-experts, with statistically significant differences observed primarily in earlier shifts (Table S1B).

### 4.2. Comparison of Setup and Cleanup Times Between Manual and TLA Workflows

To evaluate the impact of automation on pre-analytical workflow steps, setup and cleanup times were compared between manual and TLA processes across different levels of operator expertise. Setup and cleanup times were measured based on the predefined workflow activities described in the Methods section and were analyzed at the batch level.

Setup time was substantially reduced using the TLA workflow for both expert and non-expert technologists (Table 1). Among experts, the median setup time decreased from 10 min 10 s (mean: 9:49 ± 1:23; *n* = 6 batches) under manual conditions to 2 min 05 s (mean: 2:00 ± 0:18; *n* = 6) with TLA (*p* = 0.028). Similarly, among non-experts, the median setup time decreased from 13 min 37 s (mean: 14:04 ± 1:24; *n* = 6) to 2 min 20 s (mean: 2:28 ± 0:24; *n* = 6) (*p* = 0.028).

Cleanup time showed a different pattern. For expert users, median cleanup time decreased from 55 s (mean of 1:00 ± 0:29; *n* = 6) under manual processing to 25 s (mean: 0:29 ± 0:12; *n* = 6) with TLA. In contrast, for non-expert users, the median cleanup time was 60 s (mean: 1:00 ± 0:11; *n* = 6) under manual processing and 55 s (mean: 0:58 ± 0:12; *n* = 6) with TLA, with neither difference reaching statistical significance in the paired analysis (expert: *p* = 0.078; non-expert: *p* = 0.854), reflecting both the small magnitude of the cleanup-time difference and the limited statistical power of paired tests with six batches per group.

When operator experience was examined within each workflow (Table 2), expert users completed setup significantly faster than non-experts under manual conditions (*p* = 0.004), and a smaller but still significant difference persisted using TLA (*p* = 0.046). Cleanup time did not differ significantly between expert and non-expert users in the manual workflow (*p* = 0.612). However, using TLA, cleanup time was significantly shorter for expert users than for non-expert users (*p* = 0.007).

### 4.3. Total Staff Time

To assess the overall impact of automation on laboratory workflow efficiency, we evaluated total staff time (TST), defined as the sum of setup and cleanup times at the batch level (25 specimens per batch). TST provides an integrated measure of the total time required for specimen processing, capturing both pre-analytical preparation and post-analytical handling, and therefore reflects the cumulative operational burden on laboratory personnel. Similar to setup and cleanup times, TST was measured per batch and analyzed cumulatively across shifts without stratification by processing sequence (Group A or B) due to the limited number of observations per subgroup.

**Figure 2:**
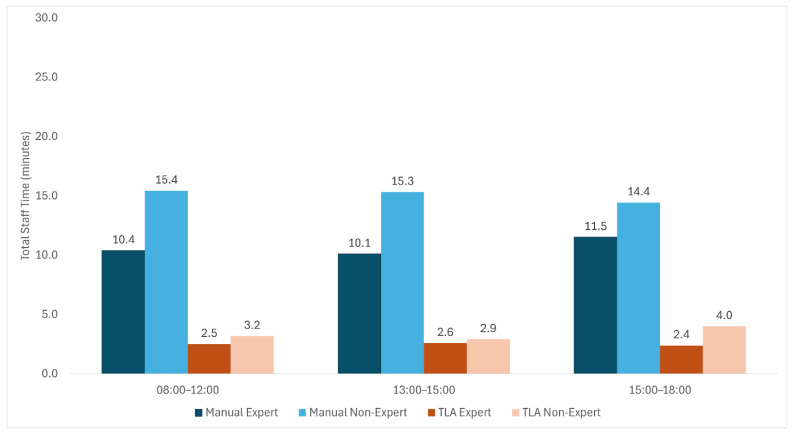
**Total staff time (TST) per batch by workflow and operator experience.** Values represent six batch-level observations per subgroup, with 25 specimens processed per batch. The median total staff time was lower with total laboratory automation (TLA) than with manual processing for both expert and non-expert technologists.

Total staff time was markedly reduced using the TLA workflow compared with manual processing. Among expert technologists, the median TST decreased from 11 min 06 s (IQR: 10:07–11:31) under manual conditions to 2 min 30 s (IQR: 2:25–2:35) with TLA. Similarly, among non-expert users, TST decreased from 14 min 37 s (IQR: 13:50–16:20) to 3 min 15 s (IQR: 3:08–3:38), reflecting a substantial reduction in total processing time across all user groups (Figure 2). When analyzed as paired batch-level measurements across shifts, this reduction was statistically significant for both expert and non-expert technologists (Wilcoxon signed-rank test: *p* = 0.028 for all comparisons).

Within the manual workflow, expert users demonstrated significantly lower TST compared to non-experts (*p* = 0.004). Using TLA, the absolute difference in TST between expert and non-expert users was substantially reduced—from approximately 3 min 31 s under manual conditions to approximately 45 s with TLA. However, this difference remained statistically significant (*p* = 0.004). Batch-level summary statistics for TST are presented in Table 1, the complete per-batch dataset is provided in Supplementary Table S3, and an overview of the workflow performance improvements is provided in Table 3.

### 4.4. Semi-Quantitative Recovery and Inoculation Consistency Across Methods

To evaluate the consistency of colony count recovery across the different inoculation workflows, a controlled comparison was performed using urine specimens with confirmed bacterial growth. Each sample was inoculated using three methods: a 1 µL manual loop (commonly used in clinical laboratories), a 10 µL manual loop, and automated inoculation using the BD Kiestra™ total laboratory automation (TLA) platform, which delivers a standardized 10 µL of inoculum via a magnetic bead-based streaking mechanism rather than a physical inoculation loop. After incubation, colony-forming unit (CFU) counts were measured semi-quantitatively and compared across methods.

Two separate experiments were conducted to assess performance across different bacterial burdens. The first experiment (Group C) included urine samples previously identified as having consistently high CFU counts, representing strong positive specimens (≥10^5^ CFU/mL). The second experiment (Group D) included randomly selected known positive samples representing a broader and more variable diagnostic range.

In Group C, colony recovery remained consistently ≥100,000 CFU/mL across all three inoculation methods, indicating comparable semi-quantitative interpretation of high-burden specimens. Despite this consistency in categorical interpretation, a statistically significant overall difference was observed across inoculation methods (*p* = 0.0256) (Table S2A). Pairwise post hoc Wilcoxon signed-rank comparisons with Bonferroni correction did not detect significant differences between any two methods after adjustment (TLA vs. 1 µL manual: adjusted *p* = 0.094; 10 µL manual vs. 1 µL manual: adjusted *p* = 0.094; TLA vs. 10 µL manual was not testable as all values were identical between methods), consistent with the comparable categorical interpretation of high-burden specimens by all three methods.

In Group D, the three inoculation methods differed in colony recovery (*n* = 10 specimens per method; Supplementary Table S2B). The median CFU counts were 80,000 CFU/mL (IQR: 42,500–87,500) with the 10 µL manual method, 40,000 CFU/mL (IQR: 25,000–50,000) with the 10 µL TLA method, and 20,000 CFU/mL (IQR: 12,000–37,500) with the 1 µL manual method. Between-method dispersion was substantially wider in the 10 µL manual data (range: 0–200,000 CFU/mL; SD: ± 61,985) than in the 10 µL TLA data (range: 0–50,000 CFU/mL; SD: ± 20,073). The Friedman test across the three methods showed a statistically significant overall difference (*p* = 0.0012). Pairwise post hoc Wilcoxon signed-rank comparisons with Bonferroni correction showed that the 10 µL manual method differed significantly from both the 10 µL TLA method (adjusted *p* = 0.047) and the 1 µL manual method (adjusted *p* = 0.012); the difference between TLA and the 1 µL manual method did not reach significance after correction (adjusted *p* = 0.375).

### 4.5. Concordance with the 1 µL Manual Reference Method

Using the same specimen sets and experimental design described previously, we evaluated the diagnostic performance of each inoculation method by applying a clinical interpretive threshold of ≥10^5^ CFU/mL to categorize samples as positive or negative. This threshold was used to calculate standard diagnostic performance metrics—sensitivity, specificity, positive predictive value (PPV), and overall accuracy—for both manual and automated workflows. As in the prior experiment, Group C comprised urine samples with known high CFU counts (strong positives), while Group D consisted of randomly selected known positive specimens, reflecting a broader diagnostic spectrum.

In Group C, manual processing by expert users yielded a high specificity (100%) and PPV (100%) but lower sensitivity (60%), resulting in an overall accuracy of 90%. Non-expert users demonstrated a higher sensitivity (80%) with identical specificity and PPV, increasing their overall accuracy to 95%. In the same Group C, the TLA platform demonstrated consistently high diagnostic performance: expert users achieved 100% sensitivity, specificity, PPV, and accuracy, while non-expert users showed a sensitivity of 80% with a specificity and PPV of 100%, yielding an overall accuracy of 95%. These results are summarized in Table 4.

In Group D, which more closely reflects routine diagnostic practice, both expert and non-expert technologists using the manual workflow achieved a sensitivity of 100%, with a slightly reduced specificity (93%) and PPV (83%), resulting in an overall accuracy of 95%. Testing Group D using TLA, both expert and non-expert users achieved a sensitivity, specificity, PPV, and accuracy of 100%, indicating complete agreement with the routine 1 µL manual reference at the ≥10^5^ CFU/mL threshold.

Diagnostic performance metrics with 95% confidence intervals are presented in Table 4; the underlying 2 × 2 contingency tables are provided in Supplementary Table S4.

## 5. Discussion

This study demonstrates that implementation of total laboratory automation (TLA) enhances laboratory efficiency and reduces operator-dependent variability; it also shows concordant semi-quantitative performance between TLA and conventional manual workflows against the standard manual reference. In particular, TLA markedly reduced total staff time (TST) and setup time across varying levels of technologist expertise, highlighting its impact on both workflow efficiency and process standardization (Figure 2; Table 1). Setup time was reduced by approximately 79–83%, while total staff time was reduced by approximately 77–78% compared with the manual workflow. These findings support the role of automation as a transformative approach in modern clinical microbiology laboratories.

Compared to manual workflows, TLA substantially accelerated specimen processing without compromising diagnostic performance, consistent with previous reports. Kritikos et al. documented significant reductions in turnaround times following TLA implementation, particularly in positive urine cultures [5]. In a separate WASPLab^® ^(Copan Italia S.p.A., Brescia, Italy) evaluation conducted during a period of substantial workforce strain, Fontana et al. reported a reduction in mean turnaround time of approximately 43.5 h for blood cultures and 20 h for biological fluid samples following TLA implementation, demonstrating that automation can maintain and even improve laboratory throughput, even under conditions of constrained staffing [17]. Similarly, Graham et al. demonstrated that automated imaging at 14 h was concordant with 24 h manual readings in 97% of cases, underscoring that diagnostic reliability is preserved despite improvements in efficiency [13]. In our study, automation reduced manual plate handling and simplified pre-analytical workflow steps, thereby streamlining specimen processing. These improvements translate into meaningful operational efficiencies, particularly in high-volume laboratory settings where manual workflows can introduce bottlenecks [18].

A key advantage of automation observed in this study is the reduction in inter-operator variability, a well-recognized limitation of manual microbiological processing. Under manual conditions, differences in performance between expert and non-expert technologists were observed, particularly in setup time, reflecting the influence of operator experience on workflow efficiency. Using TLA, these differences were substantially narrowed in absolute magnitude—from approximately 3 min 31 s under manual conditions to approximately 45 s—although they remained statistically detectable (setup: *p* = 0.046; TST: *p* = 0.004). This indicates that automation markedly reduces but does not eliminate operator-dependent variability.

In addition to improving workflow efficiency, automation contributed to more consistent semi-quantitative recovery, particularly in routine diagnostic specimens. All three inoculation methods yielded comparable categorical interpretations of high-burden specimens (Group C), where colony recovery saturated at the standard ≥10^5^ CFU/mL ceiling. In Group D, the three inoculation methods produced distinct CFU patterns. The 10 µL manual loop and the 10 µL TLA method delivered the same nominal inoculum volume, yet between-method variation was substantially wider using manual inoculation, reflecting the recognized challenges of consistent loop fill and streaking using hand inoculation [19], particularly at intermediate bacterial burdens. The lower median observed using the 1 µL manual method is consistent with a smaller inoculum volume sampling fewer organisms. By delivering a fixed 10 µL of inoculum through standardized magnetic bead-based streaking, the BD Kiestra™ TLA system reduces this volume-dependent variability and supports more consistent semi-quantitative interpretation across operators.

An important question raised by these findings is whether the lower CFU values observed under the TLA workflow in Group D could lead to misclassification of borderline specimens at the ≥10^5^ CFU/mL clinical decision threshold. Two observations argue against this. First, all Group D specimens classified as positive by the routine 1 µL manual method were correctly classified as positive using both the 10 µL TLA and 10 µL manual processing methods in the diagnostic performance experiment (Supplementary Table S4), with no false-negative classifications using TLA. Second, the lower median CFU values observed using TLA in Group D reflect tighter dispersion around the actual bacterial density of each specimen—particularly at intermediate burdens (10^4^–10^5^ CFU/mL)—rather than systematic under-recovery at the clinical threshold itself; specimens at or above ≥10^5^ CFU/mL were detected at the standard semi-quantitative ceiling using all three methods (Group C, Supplementary Table S2A). Nevertheless, careful attention to specimens with results approaching the ≥10^5^ threshold remains advisable in laboratories transitioning from manual to automated workflows, particularly during the initial validation period.

The diagnostic-performance results observed with TLA, interpreted within the limits of a proof-of-concept design, support its clinical applicability. Automation demonstrated consistently high performance across both expert and non-expert users, whereas manual workflows showed greater variability, particularly with Group C specimens. The 95% confidence intervals reported in Table 4 are wide, reflecting the proof-of-concept sample size, but the consistent pattern of TLA performance across operator groups, together with the absence of false-positive classifications using TLA in both Groups C and D (Supplementary Table S4), supports the robustness of the automated workflow within the study’s reference framework. These findings align with previous studies highlighting the reliability of automated systems across diverse user groups [13,19,20]. The ability of TLA to provide consistent diagnostic performance independent of operator expertise represents an important advantage for maintaining quality and standardization in clinical microbiology laboratories.

From an operational perspective, the reduction in total staff time observed with TLA is particularly impactful. Among expert technologists, the median TST decreased from 11 min 06 s to 2 min 30 s, while among non-expert users, it decreased from 14 min 37 s to 3 min 15 s. These reductions were statistically significant and indicate that automation can substantially improve workflow efficiency at the batch level. By decreasing the time required for specimen processing, automation enables more efficient allocation of laboratory personnel workload and increases overall throughput without requiring additional staffing. These findings are consistent with those of Kritikos et al., Zhang et al., and Khalid et al., who demonstrated that automation and optimized workflows can substantially improve laboratory efficiency, turnaround times, and reporting consistency in clinical microbiology settings [5,18,21]. Although reductions in cleanup time were more modest than those observed for setup time and total staff time, the marked decrease in setup time remained the primary driver of the overall efficiency gains. Reductions in cleanup time were observed for both expert and non-expert users, although neither reached statistical significance in the paired analysis with the available number of batch-level observations. In addition, a small but statistically significant difference in cleanup time between expert and non-expert users persisted with TLA, suggesting that minor operator-dependent variability may remain in residual manual interactions despite substantial automation-related improvements in overall workflow efficiency (Table 1 and Table 2). When scaled across high daily specimen volumes, these time savings may translate into meaningful improvements in laboratory capacity.

Furthermore, the BD Kiestra™ TLA system requires less frequent routine maintenance compared to manual workflows, with cleaning typically performed on a monthly basis according to manufacturer recommendations. This reduced maintenance burden may contribute to operational efficiency and minimize workflow interruptions.

Despite these strengths, several limitations should be acknowledged. This study was conducted in a single clinical microbiology laboratory, which may limit generalizability. Additionally, only one automation platform (BD Kiestra™ TLA) was evaluated, and performance characteristics may differ across other systems, such as Copan WASPLab. The relatively small sample size for the batch-level workflow analysis may have also limited the statistical power. Although the counterbalanced A/B processing order and the cross-shift stratification of batches were intended to distribute potential order-of-processing and time-of-day effects evenly across both workflows, residual variance attributable to learning, fatigue, or shift-related factors may persist at the individual-batch level. The diagnostic-performance comparison was designed as a proof-of-concept evaluation using twenty specimens per group and the ASM-recommended 1 µL manual loop inoculation as the within-study reference comparator, rather than an independent external reference such as molecular or mass spectrometry-based quantification. This design strictly assesses concordance between alternative inoculation methods and routine semi-quantitative references rather than diagnostic accuracy in the strict epidemiological sense, which would require an independent biological gold standard. The resulting 95% confidence intervals for sensitivity, specificity, PPV, and accuracy (Table 4) are therefore wide, particularly at the lower end of the reference positive counts, and point estimates should be interpreted accordingly. Larger-scale studies incorporating an independent external reference would be required to narrow these confidence intervals and to support broader generalizability of the diagnostic-performance findings. Finally, differences in colony distribution patterns between manual loop streaking and automated magnetic-bead streaking, as well as variability in operator familiarity with digital plate images, may influence semi-quantitative interpretation and contribute to the minor discrepancies observed between workflows. Future multicenter studies with larger sample sizes and direct comparisons across multiple automation platforms are warranted.

In conclusion, this study provides evidence that TLA improves workflow efficiency and reduces operator-dependent variability in urine culture processing, with semi-quantitative performance concordant with the standard manual reference within the limits of a proof-of-concept design. Larger-scale studies incorporating independent reference comparators will be required to confirm diagnostic-performance equivalence in broader clinical settings. As the demand for rapid, standardized, and scalable diagnostic workflows continues to grow, automation represents a valuable investment for clinical microbiology laboratories. Future work should focus on the integration of TLA with advanced diagnostic modalities, including antimicrobial susceptibility testing and molecular diagnostics, as well as evaluation of its economic impact and influence on clinical outcomes.

## Figures and Tables

**Table 1:** Comparison of setup and cleanup times per batch across workflows and user experience levels.

Process	User Group	*n*	Manual Workflow	TLA Workflow	*p*-Value
**Setup**	Expert	6	10:10 (9:06–10:29) 9:49 ± 1:23 (7:40–11:35)	2:05 (1:51–2:15) 2:00 ± 0:18 (1:30–2:15)	0.028
**Setup**	Non-Expert	6	13:37 (12:58–15:12) 14:04 ± 1:24 (12:43–15:55)	2:20 (2:15–2:25) 2:28 ± 0:24 (2:10–3:15)	0.028
**Cleanup**	Expert	6	0:55 (0:50–1:00) 1:00 ± 0:29 (0:25–1:52)	0:25 (0:20–0:34) 0:29 ± 0:12 (0:20–0:50)	0.078
**Cleanup**	Non-Expert	6	1:00 (0:53–1:00) 1:00 ± 0:11 (0:50–1:20)	0:55 (0:50–1:00) 0:58 ± 0:12 (0:50–1:20)	0.854
**TST**	Expert	6	11:06 (10:07–11:31) 10:48 ± 1:21 (8:40–12:25)	2:30 (2:25–2:35) 2:29 ± 0:07 (2:20–2:35)	0.028
**TST**	Non-Expert	6	14:37 (13:50–16:20) 15:04 ± 1:28 (13:43–16:54)	3:15 (3:08–3:38) 3:26 ± 0:29 (3:00–4:15)	0.028

Values on the first line of each cell are the median (interquartile range (IQR)) and the mean ± standard deviation (range) is on the second line. Times are reported in h:mm:ss or mm:ss format. The *n* column indicates the number of batch-level observations per cell (one batch per shift × processing-sequence combination, with 25 specimens processed per batch). Comparisons between manual and total laboratory automation (TLA) workflows within each user group were performed using the Wilcoxon signed-rank test, with two-tailed asymptotic *p*-values shown in the table. Setup time reflects the merged hands-on-plate and setup activities defined in the Methods section. The complete per-batch dataset is provided in Supplementary Table S3. Statistical significance was defined as *p* < 0.05.

**Table 2:** Comparison of expert and non-expert performance within manual and total laboratory automation (TLA) workflows.

Workflow	Process	*n*	Expert	Non-Expert	*p*-Value
**Manual**	Setup	6	10:10 (9:06–10:29) 9:49 ± 1:23 (7:40–11:35)	13:37 (12:58–15:12) 14:04 ± 1:24 (12:43–15:55)	0.004
**Manual**	Cleanup	6	0:55 (0:50–1:00) 1:00 ± 0:29 (0:25–1:52)	1:00 (0:53–1:00) 1:00 ± 0:11 (0:50–1:20)	0.612
**Manual**	TST	6	11:06 (10:07–11:31) 10:48 ± 1:21 (8:40–12:25)	14:37 (13:50–16:20) 15:04 ± 1:28 (13:43–16:54)	0.004
**TLA**	Setup	6	2:05 (1:51–2:15) 2:00 ± 0:18 (1:30–2:15)	2:20 (2:15–2:25) 2:28 ± 0:24 (2:10–3:15)	0.046
**TLA**	Cleanup	6	0:25 (0:20–0:34) 0:29 ± 0:12 (0:20–0:50)	0:55 (0:50–1:00) 0:58 ± 0:12 (0:50–1:20)	0.007
**TLA**	TST	6	2:30 (2:25–2:35) 2:29 ± 0:07 (2:20–2:35)	3:15 (3:08–3:38) 3:26 ± 0:29 (3:00–4:15)	0.004

Values on the first line of each cell are the median (interquartile range (IQR)) and the mean ± standard deviation (range) is on the second line. Times are reported in h:mm:ss or mm:ss format. The *n* column indicates the number of batch-level observations per cell. Comparisons between expert and non-expert users within each workflow were performed using the two-tailed Mann–Whitney U test, with two-sided asymptotic *p*-values given in the table. Statistical significance was defined as *p* < 0.05.

**Table 3:** Summary of workflow performance improvements achieved by total laboratory automation (TLA) compared to manual urine culture processing.

Metric	Manual Processing	TLA Processing	Improvement
**Plate Touches**	Median 3 (IQR 3–3)	0 touches	100% reduction
**Setup Time**	0:10:10–0:13:37	0:2:05–0:2:20	79–83% reduction
**Total Staff Time**	0:11:06–0:14:37	0:02:30–0:03:15	77–78% reduction
**Inter-Operator Variability**	Significant differences observed	Reduced, but not fully eliminated	Substantially reduced

This table summarizes key workflow findings. Time values are expressed in hours:minutes:seconds (hh:mm:ss). Detailed statistical comparisons are reported in the Section 4 and Table 1 and Table 2. Plate-touch values are counts per specimen, as enumerated in the Methods section (manual: labeling, inoculation, and incubator transfer; TLA: none, as these activities are performed by the automation system). The full distribution is provided in Supplementary Table S1A.

**Table 4:** Diagnostic performance by specimen group, workflow, and user experience.

Group	Workflow	User	Sensitivity (95% CI)	Specificity (95% CI)	PPV (95% CI)	Accuracy (95% CI)
**C**	Manual	Expert	60% (23–88)	100% (80–100)	100% (44–100)	90% (70–97)
**C**	Manual	Non-expert	80% (38–96)	100% (80–100)	100% (51–100)	95% (76–99)
**C**	TLA	Expert	100% (57–100)	100% (80–100)	100% (57–100)	100% (84–100)
**C**	TLA	Non-expert	80% (38–96)	100% (80–100)	100% (51–100)	95% (76–99)
**D**	Manual	Expert	100% (57–100)	93% (70–99)	83% (44–97)	95% (76–99)
**D**	Manual	Non-expert	100% (57–100)	93% (70–99)	83% (44–97)	95% (76–99)
**D**	TLA	Expert	100% (57–100)	100% (80–100)	100% (57–100)	100% (84–100)
**D**	TLA	Non-expert	100% (57–100)	100% (80–100)	100% (57–100)	100% (84–100)

Values are presented as percentages with 95% confidence intervals (CI) in parentheses, calculated using the Wilson score method. The reference comparator was the 1 µL manual loop inoculation method applied to a defined dilution series of standardized specimens (McFarland 0.5, serially diluted to ≥10^5^, 10^5^–10^4^, 10^4^–10^3^, and <10^3^ CFU/mL), consistent with American Society for Microbiology (ASM) semi-quantitative urine culture practice [10]. A clinical positivity threshold of ≥10^5^ CFU/mL was used to define reference positive specimens. Group C represents specimens with a known high bacterial load (strong positives); Group D represents randomly selected known positive specimens reflecting routine diagnostic variability. Each group comprised 20 specimens (5 reference positive, 15 reference negative) processed in parallel by manual and TLA workflows. Sensitivity = TP/(TP + FN). Specificity = TN/(TN + FP); PPV = TP/(TP + FP). Accuracy = (TP + TN)/total. PPV, positive predictive value; TLA, total laboratory automation; CI, confidence interval.

## Data Availability

The data presented in this study are available from the corresponding author upon reasonable request. The data are not publicly available due to institutional and laboratory data governance restrictions.

## Supplementary Materials

The following supporting information can be downloaded at https://www.mdpi.com/article/10.3390/diagnostics16101474/s1: Table S1. Comparison of Plate Touches Across Manual and Automated Workflows and Operator Experience Levels. Table S2. Semi-quantitative colony-forming unit (CFU) recovery across three urine inoculation methods. Table S3. Per-batch workflow timing data for all operator × workflow × shift combinations. Table S4. Two-by-two contingency tables underlying the diagnostic performance metrics reported in Table 4.

## References

[1] Mancuso G., Midiri A., Gerace E., Marra M., Zummo S., Biondo C. (2023). Urinary Tract Infections: The Current Scenario and Future Prospects. Pathogens.

[2] Somani B.K. (2025). Advancements in urinary tract infections: Understanding, prevention, diagnosis, and treatment. Ther. Adv. Infect. Dis..

[3] Santos M., Mariz M., Tiago I., Martins J., Alarico S., Ferreira P. (2022). A review on urinary tract infections diagnostic methods: Laboratory-based and point-of-care approaches. J. Pharm. Biomed. Anal..

[4] Sinawe H., Casadesus D. (2025). Urine Culture.

[5] Kritikos A., Prod’hom G., Jacot D., Croxatto A., Greub G. (2024). The Impact of Laboratory Automation on the Time to Urine Microbiological Results: A Five-Year Retrospective Study. Diagnostics.

[6] Bailey A.L., Ledeboer N., Burnham C.D. (2019). Clinical Microbiology Is Growing Up: The Total Laboratory Automation Revolution. Clin. Chem..

[7] Karah N., Rafei R., Elamin W., Ghazy A., Abbara A., Hamze M., Uhlin B.E. (2020). Guideline for Urine Culture and Biochemical Identification of Bacterial Urinary Pathogens in Low-Resource Settings. Diagnostics.

[8] Culbreath K., Piwonka H., Korver J., Noorbakhsh M. (2021). Benefits Derived from Full Laboratory Automation in Microbiology: A Tale of Four Laboratories. J. Clin. Microbiol..

[9] Dauwalder O., Landrieve L., Laurent F., de Montclos M., Vandenesch F., Lina G. (2016). Does bacteriology laboratory automation reduce time to results and increase quality management?. Clin. Microbiol. Infect..

[10] Cherkaoui A., Schrenzel J. (2022). Total Laboratory Automation for Rapid Detection and Identification of Microorganisms and Their Antimicrobial Resistance Profiles. Front. Cell Infect. Microbiol..

[11] Nam Y., Park H.D. (2025). Revolutionizing Laboratory Practices: Pioneering Trends in Total Laboratory Automation. Ann. Lab. Med..

[12] Antonios K., Croxatto A., Culbreath K. (2021). Current State of Laboratory Automation in Clinical Microbiology Laboratory. Clin. Chem..

[13] Graham M., Tilson L., Streitberg R., Hamblin J., Korman T.M. (2016). Improved standardization and potential for shortened time to results with BD Kiestra total laboratory automation of early urine cultures: A prospective comparison with manual processing. Diagn. Microbiol. Infect. Dis..

[14] Carroll K.C., Pfaller M.A., American Society for Microbiology (2019). Manual of Clinical Microbiology.

[15] Bender R., Lange S. (2001). Adjusting for multiple testing—When and how?. J. Clin. Epidemiol..

[16] Rothman K.J. (1990). No adjustments are needed for multiple comparisons. Epidemiology.

[17] Fontana C., Favaro M., Pelliccioni M., Minelli S., Bossa M.C., Altieri A., D’Orazi C., Paliotta F., Cicchetti O., Minieri M. (2023). Laboratory Automation in Microbiology: Impact on Turnaround Time of Microbiological Samples in COVID Time. Diagnostics.

[18] Khalid F., Alzahrani A.J., Mohammed H., Gamma A.K.A., Hassan M.E., Poulose C., ElSheikh A., Sumaily K., Alharbi A.A., Aldrous N.F. (2026). Impact of Total Laboratory Automation on Urine Culture Turnaround Time: A Comparative Study Between Manual Workflow and WASPLab™. Diagnostics.

[19] Strauss S., Bourbeau P.P. (2015). Impact of introduction of the BD Kiestra InoqulA on urine culture results in a hospital clinical microbiology laboratory. J. Clin. Microbiol..

[20] Moreno-Camacho J.L., Calva-Espinosa D.Y., Leal-Leyva Y.Y., Elizalde-Olivas D.C., Campos-Romero A., Alcantar-Fernandez J. (2017). Transformation From a Conventional Clinical Microbiology Laboratory to Full Automation. Lab. Med..

[21] Zhang W., Wu S., Deng J., Liao Q., Liu Y., Xiong L., Shu L., Yuan Y., Xiao Y., Ma Y. (2021). Total Laboratory Automation and Three Shifts Reduce Turnaround Time of Cerebrospinal Fluid Culture Results in the Chinese Clinical Microbiology Laboratory. Front. Cell Infect. Microbiol..

