# Supplementary material for: Total Laboratory Automation Versus Manual Processing in Urine Culture Inoculation and Interpretation: A Hospital Experience
Source: Diagnostics (Basel). 2026 May 13;16(10):1474. doi: 10.3390/diagnostics16101474 (PMC13206222; doi:10.3390/diagnostics16101474)
Supplement: Supplementary file 1 [file diagnostics-16-01474-s001.zip › diagnostics-4280209-supplementary.pdf]

Supplementary Tables

Table S1. Comparison of Plate Touches Across Manual and Automated Workflows and Operator Experience Levels

Table S1A. **Plate touches per specimen, manual vs TLA workflows, stratified by operator experience and shift**

Shift	Expert — Manual	Expert — TLA	Expert p-value	Non-expert — Manual	Non-expert — TLA	Non-expert p-value
**08–12 A**	3 (3–3); 3.00 ± 0.00; n=25	0 (0–0); n=25	< 0.001	3 (3–3); 3.20 ± 0.50; n=25	0 (0–0); n=25	< 0.001
**08–12 B**	3 (3–3); 3.00 ± 0.00; n=25	0 (0–0); n=25	< 0.001	3 (3–4); 3.28 ± 0.54; n=25	0 (0–0); n=25	< 0.001
**13–15 A**	3 (3–3); 3.00 ± 0.29; n=25	0 (0–0); n=25	< 0.001	3 (2–3); 3.04 ± 1.21; n=25	0 (0–0); n=25	< 0.001
**13–15 B**	3 (3–3); 3.04 ± 0.20; n=25	0 (0–0); n=25	< 0.001	3 (3–3); 3.12 ± 0.44; n=25	0 (0–0); n=25	< 0.001
**15-18 A**	3 (3–3); 3.04 ± 0.20; n=25	0 (0–0); n=25	< 0.001	3 (3–3); 3.12 ± 0.33; n=25	0 (0–0); n=25	< 0.001
**15-18 B**	3 (3–3); 3.08 ± 0.28; n=25	0 (0–0); n=25	< 0.001	3 (3–4); 3.28 ± 0.46; n=25	0 (0–0); n=25	< 0.001

Values are median (interquartile range, IQR) number of plate touches per specimen (count), with n = 25 specimens per shift × operator × workflow cell. Each cell reports median (IQR); mean ± SD; n in that order. Pooled across all 150 specimens per operator group, manual processing yielded median 3 (mean 3.03 ± 0.20; range 2–4) for experts and median 3 (mean 3.17 ± 0.65; range 2–8) for non-experts; TLA processing yielded zero plate touches across all 300 specimens. Plate touches were enumerated as labeling, inoculation, and incubator transfer per specimen (manual workflow); the TLA workflow involved no plate-handling interactions, with operator activity limited to specimen-tube handling captured in the setup-time metric (see Methods, Plate Touches subsection). Per-shift comparisons used the two-tailed Mann–Whitney U test; exact p-values range from 2.8×10^{-12} to 3.8×10^{-11} and are summarized as < 0.001 for display. Statistical significance was defined as $p < 0.05$.

Table S1B. Plate Touches Comparison Between Expert and Non-Expert Technologists Within the Manual Workflow Across Shifts

Shift	Expert median (IQR); mean ± SD; n	Non-expert median (IQR); mean ± SD; n	p-value
**08–12 A**	3 (3–3); 3.00 ± 0.00; n=25	3 (3–3.5); 3.20 ± 0.50; n=25	0.0457
**08–12 B**	3 (3–3); 3.00 ± 0.00; n=25	3 (3–4); 3.28 ± 0.54; n=25	0.0114
**13–15 A**	3 (3–3); 3.00 ± 0.29; n=25	3 (2–3); 3.04 ± 1.21; n=25	0.377
**13–15 B**	3 (3–3); 3.04 ± 0.20; n=25	3 (3–3); 3.12 ± 0.44; n=25	0.389
**15-18 A**	3 (3–3); 3.04 ± 0.20; n=25	3 (3–3); 3.12 ± 0.33; n=25	0.312
**15-18 B**	3 (3–3); 3.08 ± 0.28; n=25	3 (3–4); 3.28 ± 0.46; n=25	0.071

Values are median (IQR) number of plate touches per specimen (count) during manual urine culture processing, enumerated as labeling, inoculation, and incubator transfer per specimen. Comparisons between expert and non-expert technologists within each shift were performed using the two-tailed Mann–Whitney U test. Statistical significance was defined as $p < 0.05$..

Table S2. Semi-quantitative colony-forming unit (CFU) recovery across three urine inoculation methods

Table S2A. Semi-quantitative CFU recovery across inoculation methods in Group C (strong positive samples $\geq 10^5$ CFU/mL)

Inoculation Method	Median CFU/mL	IQR (CFU/mL)	Range (CFU/mL)	p-value
**TLA (10 µL bead-based inoculation)**	≥100000	≥100000 – ≥100000	≥100,000	**0.0256**
**Manual 10 µL loop**	≥100000	≥100000 – ≥100000	≥100,000	
**Manual 1 µL loop**	≥100000	≥100000 – ≥100000	80,000–≥100,000	

Group C comprised n = 10 urine specimens previously identified as having ≥10^5 CFU/mL by routine clinical culture using the standard 1 µL manual loop method. Each specimen was processed once by each of the three inoculation methods. Reported counts in this group are right-censored at the standard semi-quantitative ceiling (≥10^5 CFU/mL); means and standard deviations are therefore not reported. Comparisons across the three methods were performed using the Friedman test on within-specimen ranks. Statistical significance was defined as $p < 0.05$.

Table S2B. Semi-quantitative CFU recovery across inoculation methods in Group D (random positive samples)

Inoculation Method	n	Median CFU/mL	IQR (CFU/mL)	Mean ± SD (CFU/mL)	p-value
**TLA (10 µL bead-based inoculation)**	10	40,000	25,000–50,000	34,020 ± 20,073	**0.0012**
**Manual 10 µL loop**	10	80,000	42,500–87,500	77,020 ± 61,985	
**Manual 1 µL loop**	10	20,000	12,000–37,500	24,000 ± 19,499	

Group D comprised n = 10 randomly selected urine specimens with any positive prior routine clinical culture result, chosen to reflect the broader range of bacterial burdens encountered in routine diagnostic practice. Each specimen was processed once by each of the three inoculation methods. CFU determination in Group D was more technically demanding than in Group C:

one specimen showed no growth (NG) across all three methods; one specimen showed concordant low-burden growth at the detection floor under the 10 μL methods (200 CFU/mL by both 10 μL TLA and 10 μL manual) but no growth under the 1 μL manual method, consistent with the limited sampling sensitivity of low inoculum volumes in low-burden specimens; and several specimens in the 10^4–10^5 CFU/mL range showed substantial between-method count variation, reflecting the inherent difficulty of precise colony counting when growth is numerous but not confluent. NG values were treated as 0 CFU/mL for statistical analysis. Comparisons across the three methods were performed using the Friedman test. Statistical significance was defined as $p < 0.05$.

Supplementary Table S3. Per-batch workflow timing data for all operator × workflow × shift combinations.

Complete batch-level dataset used in the analyses reported in Tables 1A and 1B. Each row represents one batch of 25 specimens processed by a single technologist during a single shift. Shifts A and B correspond to the two processing-order permutations (A: TLA followed by manual; B: manual followed by TLA). Setup time reflects the merged hands-on-plate and setup activities defined in Methods. TST, total staff time (setup + cleanup).

Batch ID	Workflow	Operator experience	Shift	Specimens per batch	Setup time (h:mm:ss)	Cleanup time (h:mm:ss)	TST (h:mm:ss)
1	Manual	Expert	08:00–12:00 (A)	25	0:08:51	0:01:00	0:09:51
2	Manual	Expert	08:00–12:00 (B)	25	0:10:29	0:00:25	0:10:54
3	Manual	Expert	13:00–15:00 (A)	25	0:07:40	0:01:00	0:08:40
4	Manual	Expert	13:00–15:00 (B)	25	0:11:35	0:00:50	0:12:25
5	Manual	Expert	15:00–18:00 (A)	25	0:09:50	0:01:52	0:11:42
6	Manual	Expert	15:00–18:00 (B)	25	0:10:29	0:00:50	0:11:19

Batch ID	Workflow	Operator experience	Shift	Specimens per batch	Setup time (h:mm:ss)	Cleanup time (h:mm:ss)	TST (h:mm:ss)
7	Manual	Non-Expert	08:00–12:00 (A)	25	0:13:07	0:01:00	0:14:07
8	Manual	Non-Expert	08:00–12:00 (B)	25	0:15:55	0:00:50	0:16:45
9	Manual	Non-Expert	13:00–15:00 (A)	25	0:12:43	0:01:00	0:13:43
10	Manual	Non-Expert	13:00–15:00 (B)	25	0:15:34	0:01:20	0:16:54
11	Manual	Non-Expert	15:00–18:00 (A)	25	0:12:55	0:00:50	0:13:45
12	Manual	Non-Expert	15:00–18:00 (B)	25	0:14:07	0:01:00	0:15:07
13	TLA	Expert	08:00–12:00 (A)	25	0:02:15	0:00:20	0:02:35
14	TLA	Expert	08:00–12:00 (B)	25	0:01:55	0:00:30	0:02:25
15	TLA	Expert	13:00–15:00 (A)	25	0:02:15	0:00:20	0:02:35
16	TLA	Expert	13:00–15:00 (B)	25	0:02:15	0:00:20	0:02:35
17	TLA	Expert	15:00–18:00 (A)	25	0:01:50	0:00:35	0:02:25
18	TLA	Expert	15:00–18:00 (B)	25	0:01:30	0:00:50	0:02:20
19	TLA	Non-Expert	08:00–12:00 (A)	25	0:02:15	0:00:50	0:03:05
20	TLA	Non-Expert	08:00–12:00 (B)	25	0:02:25	0:00:50	0:03:15
21	TLA	Non-Expert	13:00–15:00 (A)	25	0:02:15	0:01:00	0:03:15
22	TLA	Non-Expert	13:00–15:00 (B)	25	0:02:10	0:00:50	0:03:00

Batch ID	Workflow	Operator experience	Shift	Specimens per batch	Setup time (h:mm:ss)	Cleanup time (h:mm:ss)	TST (h:mm:ss)
23	TLA	Non-Expert	15:00–18:00 (A)	25	0:03:15	0:01:00	0:04:15
24	TLA	Non-Expert	15:00–18:00 (B)	25	0:02:25	0:01:20	0:03:45

Descriptive statistics (batch-level, n = 6 per cell)

Metric	Workflow	Operator	n	Mean ± SD (h:mm:ss)	Range (h:mm:ss)
**Setup**	Manual	Expert	6	0:09:49 ± 0:01:23	0:07:40–0:11:35
**Setup**	Manual	Non-Expert	6	0:14:04 ± 0:01:24	0:12:43–0:15:55
**Setup**	TLA	Expert	6	0:02:00 ± 0:00:18	0:01:30–0:02:15
**Setup**	TLA	Non-Expert	6	0:02:28 ± 0:00:24	0:02:10–0:03:15
**Cleanup**	Manual	Expert	6	0:01:00 ± 0:00:29	0:00:25–0:01:52
**Cleanup**	Manual	Non-Expert	6	0:01:00 ± 0:00:11	0:00:50–0:01:20
**Cleanup**	TLA	Expert	6	0:00:29 ± 0:00:12	0:00:20–0:00:50
**Cleanup**	TLA	Non-Expert	6	0:00:58 ± 0:00:12	0:00:50–0:01:20
**TST**	Manual	Expert	6	0:10:48 ± 0:01:21	0:08:40–0:12:25
**TST**	Manual	Non-Expert	6	0:15:04 ± 0:01:28	0:13:43–0:16:54
**TST**	TLA	Expert	6	0:02:29 ± 0:00:07	0:02:20–0:02:35

Metric	Workflow	Operator	n	Mean ± SD (h:mm:ss)	Range (h:mm:ss)
**TST**	TLA	Non-Expert	6	0:03:26 ± 0:00:29	0:03:00–0:04:15

Descriptive statistics computed from the 24 batch-level observations above, grouped by workflow and operator experience. Each cell summarizes n = 6 independent batches (one per shift × processing-sequence combination). Values are presented as mean ± standard deviation (SD) with observed range. Medians and interquartile ranges for the same groupings are reported in Tables 1 and 2.

Supplementary Table S4. Two-by-two contingency tables underlying the diagnostic performance metrics reported in Table 4.

Each 2 × 2 contingency table summarizes the classification outcomes for one workflow (manual or TLA) and one operator group (expert or non-expert) within one specimen group (C or D). The reference comparator is the 1 µL manual loop inoculation result for the same specimen, with a clinical positivity threshold of ≥10^5 CFU/mL defining a reference-positive specimen. TP, true positive; FP, false positive; FN, false negative; TN, true negative. Each group comprised n = 20 specimens (5 reference-positive, 15 reference-negative). The sensitivity, specificity, positive predictive value, and accuracy values reported in Table 4 are derived from these counts.

Group C — Manual workflow, Expert operator

	Reference positive	Reference negative
**Test positive**	3 (TP)	0 (FP)
**Test negative**	2 (FN)	15 (TN)
**Total**	**5**	**15**

Group C — Manual workflow, Non-expert operator

	Reference positive	Reference negative
**Test positive**	4 (TP)	0 (FP)

	Reference positive	Reference negative
**Test negative**	1 (FN)	15 (TN)
**Total**	**5**	**15**

Group C — TLA workflow, Expert operator

	Reference positive	Reference negative
**Test positive**	5 (TP)	0 (FP)
**Test negative**	0 (FN)	15 (TN)
**Total**	**5**	**15**

Group C — TLA workflow, Non-expert operator

	Reference positive	Reference negative
**Test positive**	4 (TP)	0 (FP)
**Test negative**	1 (FN)	15 (TN)
**Total**	**5**	**15**

Group D — Manual workflow, Expert operator

	Reference positive	Reference negative
**Test positive**	5 (TP)	1 (FP)
**Test negative**	0 (FN)	14 (TN)
**Total**	**5**	**15**

Group D — Manual workflow, Non-expert operator

	Reference positive	Reference negative
**Test positive**	5 (TP)	1 (FP)
**Test negative**	0 (FN)	14 (TN)
**Total**	**5**	**15**

Group D — TLA workflow, Expert operator

	Reference positive	Reference negative
**Test positive**	5 (TP)	0 (FP)
**Test negative**	0 (FN)	15 (TN)
**Total**	**5**	**15**

Group D — TLA workflow, Non-expert operator

	Reference positive	Reference negative
**Test positive**	5 (TP)	0 (FP)
**Test negative**	0 (FN)	15 (TN)
**Total**	**5**	**15**
